# Protective Effects of *Bacillus subtilis* ANSB060 on Serum Biochemistry, Histopathological Changes and Antioxidant Enzyme Activities of Broilers Fed Moldy Peanut Meal Naturally Contaminated with Aflatoxins

**DOI:** 10.3390/toxins7083330

**Published:** 2015-08-21

**Authors:** Yu Fan, Lihong Zhao, Cheng Ji, Xiaoying Li, Ru Jia, Lin Xi, Jianyun Zhang, Qiugang Ma

**Affiliations:** 1 State Key Laboratory of Animal Nutrition, College of Animal Science and Technology, China Agricultural University, Beijing 100193, China; E-Mails: fanyucau@163.com (Y.F.); lihongzhao100@126.com (L.Z.); jicheng@cau.edu.cn (C.J.); li_xy8707@163.com (X.L.); jiaru.bjdk071@163.com (R.J.); jyzhang@cau.edu.cn (J.Z.); 2 Department of Animal Science, North Carolina State University, Raleigh, NC 27695, USA; E-Mail: xilin@ncsu.edu

**Keywords:** aflatoxins, aflatoxin biodegradation preparation, *Bacillus subtilis*, broiler, liver, antioxidant

## Abstract

The aim of this study was to investigate the toxic effects of aflatoxins and evaluate the effectiveness of *Bacillus subtilis* ANSB060 in detoxifying aflatoxicosis in broilers. A total of 360 one-week-old male broilers (Ross 308) were assigned to six dietary treatments for five weeks. The treatment diets were: C0 (basal diet); C1.0 (C0 + 1.0 g *B. subtilis* ANSB060/kg diet); M0 (basal diet formulated with moldy peanut meal); M0.5, M1.0 and M2.0 (M0 + 0.5, 1.0 and 2.0 g *B. subtilis* ANSB060/kg diet, respectively). The contents of aflatoxin B_1_, B_2_, G_1_ and G_2_ in the diets formulated with moldy peanut meal were 70.7 ± 1.3, 11.0 ± 1.5, 6.5 ± 0.8 and 2.0 ± 0.3 µg/kg, respectively. The results showed that aflatoxins increased (*p* < 0.05) serum aspartate transaminase activity, decreased (*p* < 0.05) serum glutathione peroxidase activity, and enhanced (*p* < 0.05) malondialdehyde contents in both the serum and liver. Aflatoxins also caused gross and histological changes in liver tissues, such as bile duct epithelium hyperplasia, vacuolar degeneration and lymphocyte infiltration. The supplementation of ANSB060 reduced aflatoxin levels in the duodenum and counteracted the negative effects of aflatoxins, leading to the conclusion that ANSB060 has a protective effect against aflatoxicosis and this protection is dose-related.

## 1. Introduction

Aflatoxins are a class of potent mycotoxins produced mainly by *Aspergillus flavus*, *Aspergillus parasiticus*, and occasionally by other *Aspergillus* species [1]. Aflatoxins occur widely in naturally contaminated animal feeds and human foods, such as peanuts, maize, distiller’s dried grains with solubles (DDGS), silage, oilseeds, milk, cheese, fruit juice, and other agricultural feed or food [2,3]. Aflatoxins constitute a great threat to the health of animals and humans due to their teratogenic, carcinogenic, mutagenic, and immunosuppressive effects [4,5]. Additionally, in terms of the livestock industry, aflatoxins cause huge economic loss by retarding animal growth, increasing feed consumption, and reducing meat production [6,7]. Among the various types of aflatoxins, aflatoxin B_1_ (AFB_1_) is known to be the most biologically active component.

It is reported that the liver is the main target organ for aflatoxins. Long-term intake of feeds contaminated with aflatoxins results in negative effects on the liver, such as hepatic cell and tissue injury [6], as well as gross and microscopic abnormalities [8]. Abdel-Wahhab and Aly [9] reported that aflatoxins caused membrane damage in rat liver through increased lipid peroxidation. According to Özen *et al.* [10], AFB_1_ increased the malondialdehyde (MDA) level, and induced vacuolar degeneration, necrosis, and bile duct hyperplasia in chicken liver. These deteriorations were confirmed to increase susceptibility to infectious disease and mortality in animals [11]. Moreover, residues of aflatoxins in the tissues, milk, and eggs of animals are a potential hazard to human health [12]. Researchers revealed the relationship between aflatoxin exposure and high incidence of human liver cancer in various areas of Asia and Africa [13]. Aflatoxins can act synergistically with the hepatitis B virus to enhance the risk of hepatocellular carcinoma [14]. Since the occurrence of aflatoxins in feeds and foods is generally at low concentrations [2,3], there is interest in studying the effects of long-term exposure to low levels of aflatoxins on animals and humans.

Numerous physical, chemical and biological methods have been proposed to detoxify or inactivate aflatoxins in contaminated feedstuffs. Among them, the use of biological methods, using microorganisms and their metabolites to eliminate aflatoxins, can be a highly promising approach owing to its specific, efficient, and environmentally sound detoxification [15]. Some microbes, including fungal and bacterial isolates, such as *Flavobacterium aurantiacum* [16], *Stenotrophomonas Maltophilia* [5], *Myxococcus fulvus* [17], and *Aspergillus niger* [18], were reported to effectively biodegrade aflatoxins *in vitro*. However, little is known about their efficiency in the biodegradation of aflatoxins and effect on aflatoxicosis *in vivo*. Our lab screened a strain of probiotic bacteria *Bacillus subtilis* ANSB060 from the fish gut, which can be directly applied in the feedstuffs and feeds [19]. This strain possessed strong degradation ability against aflatoxins (up to 81.5%). Particularly, the cell-free culture supernatant showed effective aflatoxin-degrading activity (up to 78.7%), suggesting that *B. subtilis* ANSB060 can detoxify aflatoxins via biotransformation rather than binding or absorbing aflatoxins to cell walls. In addition, it exhibited antimicrobial activity and high resistance to the simulated gut environment. Because *B. subtilis* is generally recognized as safe, it is feasible for *B. subtilis* ANSB060 to be applied in the feeds and test its protective effects against aflatoxicosis in animals. The effects of supplementation of *B. subtilis* ANSB060 in the aflatoxin-contaminated diets on improving growth performance in layers and broilers have been verified well in our previous studies [7,20]. However, the role of dietary *B. subtilis* ANSB060 in protecting hepatic structure and function from aflatoxins in broilers has not been assessed so far.

The objective of this study was to examine the toxic effect of aflatoxins and protective efficacy of *B. subtilis* ANSB060 on serum biochemistry, liver histopathology, serum and liver antioxidant enzymes activities, and aflatoxin concentrations in the duodenal content of broilers exposed to feed naturally contaminated with aflatoxins.

## 2. Results

### 2.1. Determination of Mycotoxin Content

The naturally moldy peanut meal used in the diets was mainly contaminated with aflatoxins. The concentrations of AFB_1_, AFB_2_, AFG_1_ and AFG_2_ were determined to be 70.7 ± 1.3, 11.0 ± 1.5, 6.5 ± 0.8 and 2.0 ± 0.3 µg/kg diet, respectively, in the treatment diets of M0, M0.5, M1.0 and M2.0. The content of zearalenone (ZEA) in the moldy diets (M0, M0.5, M1.0 and M2.0) was only 4.1 ± 0.3 µg/kg diet. Other mycotoxins, namely deoxynivalenol (DON) and ochratoxin A (OTA), were not detected in the diets of M0, M0.5, M1.0 and M2.0. The concentrations of all mycotoxins analyzed in the diets of C0 and C1.0 were below the detection limits under current analytical conditions. The detection limits for AFB_1_, AFB_2_, AFG_1_, AFG_2_, DON, ZEA and OTA were 1.0, 0.7, 3.7, 1.8, 60, 3.0 and 0.5 µg/kg diet, respectively. 

### 2.2. Organ Weights and Serum Biochemistry

No significant differences were observed for the weights of organs (heart, liver, spleen, bursa of fabricius and thymus) among treatments (*p* > 0.05; Table 1). However, the aspartate aminotransferase (AST) activity measured in the chickens fed diet M0 was increased by 37% (*p* < 0.001; Table 2) as compared with the chickens fed diet C0. The AST activity in group C1.0 was not different from group C0 (*p* > 0.05). Supplementation of *B. subtilis* ANSB060 to diets contaminated with aflatoxins decreased the AST activity and the decrease magnitude was associated with the level of *B. subtilis* ANSB060 supplemented. Polynomial orthogonal contrasts showed that both linear and quadratic responses to *B. subtilis* ANSB060 supplementation in the aflatoxin-contaminated diet were significant (*p* < 0.001). The AST activity measured in chickens fed diet M2.0 had no difference from diet C0. No differences were detected for all the other enzymes among the treatment groups (*p* > 0.05).

**Table 1:** Effects of *B. subtilis* ANSB060 on the relative organ weights (g/kg of body weight (BW)) of broilers fed moldy peanut meal naturally contaminated with aflatoxins ^1^.

Item	C0	C1.0	M0	M0.5	M1.0	M2.0	SEM	*p*-Value		
								F-test	Linear	Quadratic
BW, kg	2.353 ^a^	2.344 ^a^	2.257 ^b^	2.294 ^ab^	2.320 ^ab^	2.331 ^a^	0.010	0.049	0.019	0.571
Heart	3.33	3.15	3.22	3.10	3.30	3.14	0.113	0.652	0.914	0.853
Liver	22.90	22.80	23.93	23.23	23.52	23.76	0.618	0.749	0.937	0.456
Spleen	1.08	1.20	1.11	1.14	1.22	1.16	0.073	0.790	0.499	0.544
Bursa of fabricius	0.51	0.54	0.61	0.62	0.61	0.57	0.038	0.195	0.417	0.518
Thymus	1.45	1.35	1.72	1.56	1.83	1.74	0.203	0.541	0.722	0.878

^1^ Data are expressed as group mean values (*n* = 12); BW, body weight; SEM, standard error of the mean; Linear, linear effect of the graded concentration of *B. subtilis* ANSB060 supplementation in the moldy diet; Quadratic, quadratic effect of the graded concentration of *B. subtilis* ANSB060 supplementation in the moldy diet. ^a−b^ Means with different superscripts in each row differ significantly (*p* < 0.05).

**Table 2:** Effects of *B. subtilis* ANSB060 on serum biochemical parameters of broilers fed moldy peanut meal naturally contaminated with aflatoxins ^1^.

Item	C0	C1.0	M0	M0.5	M1.0	M2.0	SEM	*p*-Value		
								F-test	Linear	Quadratic
TP, g/L	25.88	25.25	23.87	23.74	23.91	24.04	0.968	0.524	0.876	0.894
ALT, U/L	2.08	2.20	1.97	2.19	2.03	1.83	0.423	0.990	0.766	0.611
AST *, U/L	34.25 ^b^	36.22 ^ab^	47.14 ^a^	48.29 ^a^	41.45 ^ab^	31.84 ^b^	0.853	<0.001	<0.001	<0.001
ALP, U/L	1419.5	1425.8	1517.0	1489.4	1440.4	1437.6	49.291	0.678	0.203	0.803

^1^ Data are expressed as group mean values (*n* = 12); TP, total protein; ALT, alanine transaminase; AST, aspartate aminotransferase; ALP, alkaline phosphatase; SEM, standard error of the mean; Linear, linear effect of the graded concentration of *B. subtilis* ANSB060 supplementation in the moldy diet; Quadratic, quadratic effect of the graded concentration of *B. subtilis* ANSB060 supplementation in the moldy diet; ^a−b^ Means with different superscripts in each row differ significantly (*p* < 0.05); * Contrast of M0 *versus* M0 supplemented with *B. subtilis* ANSB060 (M0 *vs.* M0.5+M1.0+M2.0) (AST, *p* < 0.001).

### 2.3. Serum and Liver Antioxidant Enzyme Activities

Serum and hepatic total superoxide dismutase (SOD) activities were not affected (*p* > 0.05) by dietary treatments.

The serum activity of glutathione peroxidase (GSH-Px) measured in chickens under M0 treatment was on average 7% lower (*p* < 0.05) than that in the chickens under C0 and C1.0 treatments but was enhanced with the supplementation of *B. subtilis* ANSB060 at the rate of 2 g/kg of M0 diet (Table 3). This enhancement was linear with the increase of *B. subtilis* ANSB060 supplemented in the moldy diets (*p* < 0.05). No difference in GSH-Px activity was detected between group M2.0 and C0. The serum MDA content was increased by 46% on average in chickens fed diet M0 compared with the chickens fed diets C0 and C1.0 (*p* < 0.001). Supplementation of *B. subtilis* ANSB060 at 2 g/kg of M0 diet decreased serum MDA content. A negative linear response of serum MDA level to the amount of *B. subtilis* ANSB060 was also observed (*p* = 0.001). No difference was tested in the serum MDA between chickens fed diets M2.0 and C0.

Like the serum MDA level, the level of liver MDA in group M0 was on average 18% higher (*p* < 0.05) than that in C0 and C1.0, and supplementing *B. subtilis* ANSB060 to the moldy diets restored the MDA content under treatment M2.0. The hepatic MDA content declined linearly (*p* < 0.05) with increase in the levels of *B. subtilis* ANSB060. Chickens fed diets of M1.0 and M2.0 had similar MDA content in the liver (*p* > 0.05) as chickens fed a diet of C0.

**Table 3:** Effects of *B. subtilis* ANSB060 on serum and liver antioxidant index of broilers fed moldy peanut meal naturally contaminated with aflatoxins ^1^.

Item	C0	C1.0	M0	M0.5	M1.0	M2.0	SEM	*p*-Value		
								F-test	Linear	Quadratic
Serum	-	-	-	-	-	-	-	-	-	-
SOD, U/mgprot	119.33	116.01	110.86	113.19	115.01	117.75	2.943	0.404	0.093	0.943
GSH-Px, U/mgprot	491.62 ^a^	492.04 ^a^	457.25 ^c^	468.12 ^bc^	469.56 ^bc^	479.75 ^ab^	6.686	0.005	0.027	0.960
MDA *, nmol/mgprot	16.01 ^c^	17.11 ^c^	24.21 ^a^	22.64 ^a^	21.14 ^ab^	18.63 ^bc^	1.025	<0.001	0.001	0.656
Liver	-	-	-	-	-	-	-	-	-	-
SOD *, U/mgprot	297.15	296.83	275.48	293.11	291.32	293.66	6.899	0.332	0.106	0.280
GSH-Px, U/mgprot	72.483	72.339	63.157	67.315	67.640	71.783	2.589	0.095	0.031	0.997
MDA ^*^, nmol/mgprot	0.218 ^c^	0.225 ^bc^	0.262 ^a^	0.245 ^ab^	0.240 ^abc^	0.227 ^bc^	0.008	0.007	0.004	0.824

^1^ Data are expressed as group mean values (*n* = 12); SOD, total superoxide dismutase; GSH-Px, glutathione peroxidase; MDA, malondialdehyde; SEM, standard error of the mean; Linear, linear effect of the graded concentration of *B. subtilis* ANSB060 supplementation in the moldy diet; Quadratic, quadratic effect of the graded concentration of *B. subtilis* ANSB060 supplementation in the moldy diet; ^a−c^ Means with different superscripts in each row differ significantly (*p* < 0.05); * Contrast of M0 *versus* M0 supplemented with *B. subtilis* ANSB060 (M0 *vs.* M0.5+M1.0+M2.0) (serum MDA, *p* = 0.009; hepatic SOD, *p* = 0.049; hepatic MDA, *p* = 0.011).

### 2.4. Histopathology

**Figure 1:**
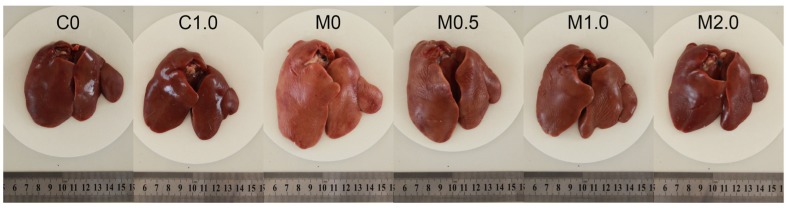
Representative livers from broilers (42 days old) fed different diets. C0, the negative control diet; C1.0, the negative control diet plus 1.0 g *B. subtilis* ANSB060 /kg diet; M0, the aflatoxin-contaminated diet; M0.5, the aflatoxin-contaminated diet plus 0.5 g *B. subtilis* ANSB060/kg diet; M1.0, the aflatoxin-contaminated diet plus 1.0 g *B. subtilis* ANSB060/kg diet; M2.0, the aflatoxin-contaminated diet plus 2.0 g *B. subtilis* ANSB060/kg diet.

Macroscopically, the livers from the birds fed a diet of M0 were slightly enlarged and pale in color compared to those from the birds under the control treatment (Figure 1). The livers of birds in group C1.0 had an appearance similar to those in group C0. Supplementation of B. subtilis ANSB060 in the diets containing aflatoxins could ameliorate these changes. As presented in Figure 1, the intensity of the amelioration appeared to increase in a dose related manner.

**Figure 2:**
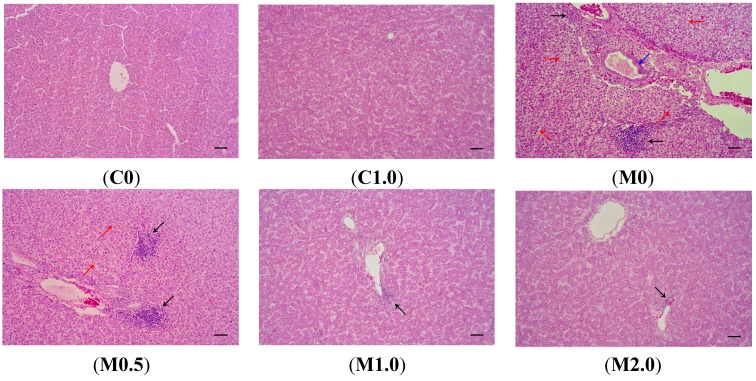
Representative photomicrographs (optical microscopy) of hematoxylin and eosin-stained broiler liver sections from different treatments. (**C0**) normal histological structure of liver lobule, central vein and hepatocytes are observed in broilers fed negative control diet; (**C1.0**) normal hepatocytes are present in broilers fed negative control diet plus 1.0 g *B. subtilis* ANSB060 /kg diet; (**M0**) obvious liver lesions such as bile duct epithelium hyperplasia (blue arrow), lymphocyte infiltration in hepatocytes and portal tract (black arrow), and vacuolar degeneration in hepatocytes (red arrow) are observed in broilers fed diet contaminated with aflatoxins; (**M0.5**) lymphocyte infiltration in hepatocytes (black arrow) and less vacuolar degeneration in hepatocytes (red arrow) are present in broilers fed aflatoxin-contaminated diet plus 0.5 g *B. subtilis* ANSB060/kg diet; (**M1.0**) less lymphocyte infiltration in hepatocytes (black arrow) is observed in broilers fed aflatoxin-contaminated diet plus 1.0 g *B. subtilis* ANSB060/kg diet; (**M2.0**) the least lymphocyte infiltration in hepatocytes (black arrow) is present in broilers fed aflatoxin-contaminated diet plus 2.0 g *B. subtilis* ANSB060/kg diet. Scale bar = 50 μm.

Figure 2 shows the photomicrographs of hematoxylin and eosin-stained liver sections of the birds from different dietary treatments. There were no visible lesions in the livers of the birds from groups C0 and C1.0. Livers from the birds consuming diet M0 showed significant lesions such as bile duct epithelium hyperplasia, vacuolar degeneration in hepatocytes, and lymphocyte infiltration in hepatocytes and portal tract. The supplementation of *B. subtilis* ANSB060 to diet M0 partially decreased the severity of these lesions. As the amount of *B. subtilis* ANSB060 increased in the diet, the amelioration on the lesions was more apparent, especially under the M2.0 treatment.

### 2.5. Levels of Aflatoxins Recovered from Duodenal Content

**Figure 3:**
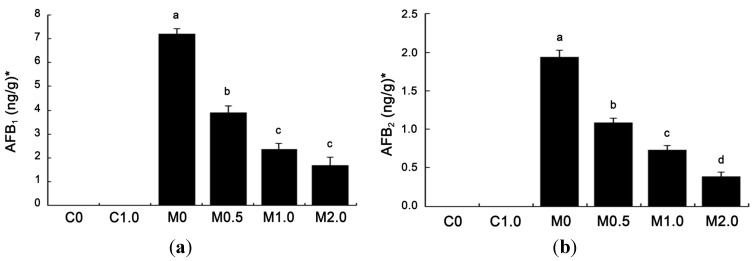
Effects of *B. subtilis* ANSB060 on AFB_1_ (**a**) and AFB_2_ (**b**) recovered from duodenal content of broilers fed moldy peanut meal naturally contaminated with aflatoxins. Values are expressed as mean ± SE, *n* = 6. Columns with different letters are significantly different (*p* < 0.05). * AFB_1_ and AFB_2_ were not detected in the duodenum of broilers under C0 and C1.0 treatments; AFB_1_ and AFB_2_ showed linear and quadratic responses to the increasing addition of *B. subtilis* ANSB060 in the moldy diet (*p* < 0.05).

The amounts of aflatoxins recovered from duodenal content are given in Figure 3. Neither AFG_1_ nor AFG_2_ was detected in duodenal contents (detection limit, 1.00 ng/g for AFG_1_ and 0.50 ng/g for AFG_2_). AFB_1_ and AFB_2_ were not detected in the duodenum of broilers fed diets C0 and C1.0 (detection limit, 0.30 ng/g for AFB_1_ and 0.20 ng/g for AFB_2_). Among other treatments, AFB_1_ level was the highest in group M0 at 7.21 ng/g freeze-dried matter. Compared to group M0, supplementing *B. subtilis* ANSB060 to contaminated diets (M0.5, M1.0 and M2.0) markedly reduced the AFB_1_ concentration by 46.05% (3.89 ng/g), 67.13% (2.37 ng/g) and 76.70% (1.68 ng/g), respectively. The amount of AFB_2_ in all groups had a similar trend as that of AFB_1_. The level of AFB_2_ in group M0 was at 1.94 ng/g, and reduced by 43.81% (1.09 ng/g), 62.37% (0.73 ng/g) and 79.90% (0.39 ng/g) (*p* < 0.05) with the increase of supplementing *B. subtilis* ANSB060 to groups M0.5, M1.0, and M2.0, respectively. Both the AFB_1_ and AFB_2_ concentrations showed linear and quadratic responses to the increasing addition of *B. subtilis* ANSB060 to the M0 diet.

## 3. Discussion

### 3.1. Toxicity of Aflatoxins

The liver is considered the primary target organ for aflatoxins because aflatoxins are predominantly accumulated and metabolized in the liver after absorption. It has been reported that 1000 μg AFB_1_/kg of diet could cause a significant increase in the relative liver weight of broilers [21]. The enhancement of liver weight is perhaps due to an inhibition of lipid transport and lipid accumulation in the liver [22]. However, the changes in liver weight appear to be associated with the aflatoxin level in the diet. In the present study, no significant difference in liver weight was found when broilers were exposed to aflatoxins at a total level of 90.2 μg/kg of diet (AFB_1_ = 70.7 μg/kg, 7% of the reported value). Similar results were also noticed in previous reports, in which the liver weight of broilers did not change when the dietary aflatoxin concentration was at 50–100 μg/kg of diet [8,23]. Therefore, we infer that perhaps this variable, the weight of the liver, is not sensitive to low-dose aflatoxins.

The serum activities of AST, alanine transaminase (ALT), and alkaline phosphatase (ALP) have been recognized as sensitive serological indicators in the impairment of the hepatic tissues and biliary system, and the serum level of total protein (TP) is the indicator of protein synthesis [24]. Therefore, in our study, the increased serum AST activity observed in the chickens fed diets containing aflatoxins indicates that at least certain damage occurred in the liver. This is because AST, originally located in the cytoplasm, is released into the blood system only when hepatic structural integrity is affected. Although the AST activity was significantly increased, we did not detect significant alterations in the levels of serum TP, ALT and ALP in the chickens that received aflatoxins. This was consistent with a previous report in which broilers were exposed to diets containing AFB_1_ at a level of 82.4 μg/kg of diet [25]. However, some reports demonstrated that a high level of AFB_1_ (2500 μg/kg) significantly decreased serum TP content and/or increased serum ALT and ALP activities in animals [24,26,27]. The differences among the results suggest that changes in serum TP, ALT, and ALP are also closely related with the level of aflatoxins in the diet.

Although no changes in serum TP, ALT, and ALP levels were noted, discoloration and swelling of the liver, along with bile duct epithelium hyperplasia, vacuolar degeneration in hepatocytes, and lymphocyte infiltration in hepatocytes and portal tract, were observed in birds fed the diets containing aflatoxins. The histopathological observations from the liver tissues were similar to those reported in the literature [28]. The gross and histopathological lesions in the livers of birds under aflatoxin diets as noted by us are in agreement with the observed serum AST alteration.

Aflatoxins generate intracellular reactive oxygen species (ROS), such as superoxide anion, hydrogen peroxide and hydroxyl radicals, during its metabolic processing in the liver. SOD and GSH-Px are the crucial antioxidant enzymes scavenging ROS in cells. Oxidative stress occurs when the level of ROS exceeds the tolerance capacity of the cellular antioxidant defense system. As end product of lipid peroxidation, MDA contents in blood and tissues increase following the occurrence of oxidative stress. Overall, our observations regarding the reduction of GSH-Px activity in serum and increased MDA level in both the serum and liver in the birds fed the M0 diet are consistent with the previous reports on broilers exposed to aflatoxins [25,29]. These data imply that feeding diet formulated with peanut meal naturally contaminated with aflatoxins induced oxidative damage and lipid peroxidation in chickens, which might be the main cause of liver tissue damage and serum biochemical alterations.

### 3.2. Effect of B. subtilis ANSB060

Due to the frequent occurrence of aflatoxin and its potent toxicity to humans and animals, seeking an effective detoxification strategy has become the subject of numerous studies. Biological methods to degrade aflatoxins are important in this respect. The *B. subtilis* ANSB060 used in the present study was originally isolated from fish gut in our laboratory, and it has already been shown to possess a strong capability to biodegrade aflatoxins (up to 81.5%) [19]. *B. subtilis* ANSB060 can be fed to animals in a safe way, as demonstrated by the results on broiler’s growth performance and meat quality [7]. In this study, chickens fed uncontaminated diet supplemented with *B. subtilis* ANSB060 at 1.0 g/kg showed no differences for all the tested indices from the control, suggesting that the supplementation of *B. subtilis* ANSB060 in feed has no negative impact on the liver function of chickens. Moreover, supplementation of *B. subtilis* ANSB060 to aflatoxin-contaminated diets was found to counteract the oxidative damage, histopathological lesions, and serum biochemical changes induced by the aflatoxins in a dose-related manner, and 2.0 g/kg *B. subtilis* ANSB060 provided maximum protection. These results are in agreement with our previous study on dwarf layers [20]. Similar effects of other bacteria on aflatoxicosis have also been reported in the literature, but the protective action is primarily based on the absorption or binding to the bacterial cells. For instance, strains of *Lactobacillus casei* and *Lactobacillus reuteri* successfully alleviated the liver damage of rats fed aflatoxins-contaminated diet via their ability to bind aflatoxins in the gastrointestinal tract [30]. Bagherzadeh Kasmani *et al.* [27] also found that changes in serum biochemistry of Japanese quails associated with AFB_1_ contamination could be ameliorated by binder strain *Brevibacillus laterosporus*. Unlike these bacteria, *B. subtilis* ANSB060 biodegrades aflatoxins directly without inducing pollution of the environment [19]. Therefore, the application of *B. subtilis* ANSB060 in feedstuffs may have a more bright and valuable future.

In our previous study, the results implied that the activity of aflatoxin degradation was mainly in the culture supernatant of *B. subtilis* ANSB060 rather than its cells or cell extracts [30]. After heating and proteinase K treatment, the activity of aflatoxins degradation was decreased by 56% and 73%, indicating that the function of *B. subtilis* ANSB060 to detoxify aflatoxins may be due to biodegradation. In the present study, the supplementation of *B. subtilis* ANSB060 in the contaminated diet resulted in a linear decrease in recovery of aflatoxins from duodenal contents. These results are consistent with our previous study, in which the residual levels of aflatoxins in the liver were significantly reduced by *B. subtilis* ANSB060 supplementation [7]. These findings imply that *B. subtilis* ANSB060 could detoxify aflatoxins in animal gastrointestinal tracts and reduce the amount of aflatoxins absorbed into the body, consequently preventing the detrimental effects of aflatoxins to the animals and environment. However, the specific biotransformation mechanism of *B. subtilis* ANSB060 to detoxify aflatoxins is being studied and still unclear. Further studies are warranted in this area.

## 4. Experimental Section

### 4.1. Animals, Design and Diets

A total of 360 one-day-old male broiler chickens (Ross 308) were purchased from a commercial hatchery. After an acclimatization period of seven days, broilers (body weight = 162.0 ± 0.4 g) were assigned randomly into six treatments with six replicates per treatment and ten birds per replicate. The diets for the treatments were: C0 (the negative control, a basal diet containing 21% normal peanut meal); C1.0 (the negative control supplemented with 1.0 g *B. subtilis* ANSB060/kg diet); M0 (the positive control, the basal diet containing 21% moldy peanut meal substituting for the normal peanut meal); and M0.5, M1.0 and M2.0 (the positive control supplemented with 0.5, 1.0 and 2.0 g *B. subtilis* ANSB060/kg diet). The basal diet (Table 4) was formulated to meet the nutrient requirements of the National Research Council (1994). After preparing the diet, two samples of feed from each treatment were analyzed to ensure mycotoxin concentrations in the experimental diets. The concentrations of aflatoxins (including AFB_1_, AFB_2_, AFG_1_ and AFG_2_), DON, ZEA and OTA in the diets were determined using high performance liquid chromatography (HPLC) as described by Binder *et al.* [2]. Briefly, 25 g of milled samples were well-mixed with 100 mL of methanol-water (80:20, vol/vol) for aflatoxins; water for DON; acetonitrile-water (70:30, vol/vol) for ZEA; methanol-water (60:40, vol/vol) for OTA. The mixtures were shaken vigorously for 1 h. The extract was filtered, and the filtrate was cleaned up through an immunoaffinity column (Vicam, Milford, MA, USA) before HPLC (Shimadzu LC-10 AT, Shimadzu, Tokyo, Japan) determination. An aflatoxin biodegradation preparation consisting mainly of *B. subtilis* ANSB060 was produced by industrial fermentation and dry-processing technologies. The viable count of *B. subtilis* ANSB060 in the aflatoxin biodegradation preparation was more than 1 × 10^9^ CFU/g.

**Table 4:** Basal diet formulations and nutritional contents.

Ingredients	Percentage (%)	Nutrition component	Content
Maize	57.70	Crude protein, %	21.48
Extruded-soybean	6.00	Metabolisable energy, MJ/kg	12.60
Soybean meal	8.20	Calcium, %	0.99
Peanut meal	21.00	Total phosphorus, %	0.65
Limestone	1.37	Available phosphorus, %	0.43
Calcium hydrophosphate	1.80	Methionine, %	0.62
Salt	0.30	Methionine + Cystine, %	0.91
Soybean oil	2.00	Lysine, %	1.15
Lysine [98.5%]	0.47	Tryptophan, %	0.21
DL-methionine	0.36	Threonine, %	0.81
Threonine	0.19	-	-
Vitamin premix ^1^	0.03	-	-
Choline chloride	0.10	-	-
Mineral premix ^2^	0.30	-	-
Zeolite powder	0.18	-	-
Total	100.00	-	-

^1^ Provided per kilogram of diet: 12,000 IU vitamin A; 3,000 IU cholecalciferol; 7.5 IU vitamin E; 1.5 mg vitamin K_3_; 0.6 mg thiamine; 4.8 mg riboflavin; 1.8 mg pyridoxine; 9 μg vitamin B_12_; 150 μg folic acid; 10.5 mg niacin. ^2^ Provided per kilogram of diet: 7.5 mg calcium pantothenate; 30 mg Fe as ferrous sulfate; 8.0 mg Cu as copper sulfate; 120 mg Mn as manganous oxide; 100 mg Zn as zinc sulfate; 0.30 mg Se as sodium selenite; 0.70 mg I as ethylenediamine dihydroiodide.

The broilers were given humane care in compliance with the guidelines of the Animal Welfare Committee of China Agricultural University. All broilers were raised in wire cages in a three-level battery. Broilers were exposed to 24-h continuous lighting for the first three days and 23L: 1D (23 h of light and 1 h of darkness) from four days of age onward. The temperature was initially maintained at 30 °C for the first week and gradually decreased to 21 °C until 24 days and maintained at 21 °C thereafter. The relative humidity was maintained at between 65% and 70%. Ventilation was controlled by negative pressure using fans. Feed and water were provided *ad libitum* via tube feeders and nipple drinkers during the entire experimental period.

### 4.2. Serum Biochemistry and Organ Weights

At 42 days of age, two birds close to the average weight were selected from each replicate. After the birds were fasted for 12 h, blood samples were collected in tubes without anticoagulant by puncture of the wing vein. The samples were centrifuged at 1000× *g* at 4 °C for 10 min, and the serum was separated and stored at −70 °C until biochemical analysis. The TP content, along with the ALT, AST, and ALP activities were determined using commercial kits (Nanjing Jiancheng Bioengineering Institute, Nanjing, China) with the colorimetric method following the kit instructions. After taking blood samples, the birds were euthanized humanely by cervical dislocation, and the liver, heart, spleen, bursa of fabricius, and thymus were removed and weighed immediately.

### 4.3. Serum and Liver Antioxidant Enzyme Activities Assays

Within 1 h postmortem, the liver samples (the tip of the left lobe) were removed and washed in ice-cold physiological saline. Approximately 0.5 g of liver was homogenized in 4.5 mL ice-cold physiological saline using an Ultra-Turrax (T8, IKA-Labortechnik, Staufen, Germany). The homogenate was then centrifuged at 1200× *g* at 4 °C for 10 min. The supernatant was collected and stored in a freezer at −70 °C for the following analysis. The activities of SOD and GSH-Px, and contents of MDA in the serum and hepatic supernatants were measured using commercial kits (Nanjing Jiancheng Bioengineering Institute, Nanjing, China) according to the kit instructions.

### 4.4. Histopathological Measurements

Part of the liver sample (the tip of the right lobe) from the birds was also taken and fixed in 10% neutral-buffered formalin solution. The fixed tissue was dehydrated in graded alcohol, cleared in xylene, and embedded in paraffin. Thin sections (3 to 5 μm) were sliced and then stained with hematoxylin and eosin for histopathological examination using optical microscopy (Olympus Optical Company, Tokyo, Japan) as described by Bancroft and Gamble [31].

### 4.5. Aflatoxins Recovered from Duodenal Content Measurements

At 42 days of age, another two birds (without fasting) with the average body weight in each replicate were selected and euthanized humanely by cervical dislocation. The duodenal contents (from ventriculus to pancreo-biliary ducts) of the two birds from the same replicate were collected in the same tube. To remove all the content, the duodenum was rinsed with 15 mL phosphate buffered saline (PBS, pH = 7.4).

The analysis of aflatoxin levels recovered from duodenum was carried out according to the methods of the Association of Official Analytical Chemists (AOAC, 2000). The sample was cleaned up before HPLC determination. Specifically, 0.5 g of the freeze-dried duodenal contents were blended in 4 mL of methanol-water (80:20, vol/vol) for 3 min, and the resulted homogenate was centrifuged at 2500× *g* for 5 min. The supernatant (2 mL) obtained from the centrifugation was diluted with 8 mL of PBS and passed through an immunoaffinity column (Vicam, Milford, MA, USA). Aflatoxins were eluted from the column with 1.0 mL methanol into a clear glass tube, and the methanol was evaporated to dryness under a gentle stream of nitrogen. The residue was dissolved in the HPLC mobile phase for analysis. The HPLC system (Shimadzu LC-10 AT, Shimadzu, Tokyo, Japan) was equipped with a reverse phase column (DIKMA, C18, 5 μm, 15 cm × 4.6 cm ID), a post-column photochemical derivation (Aura Industries, Staten Island, NY, USA), and a fluorescence monitor (Shimadzu RF-20A, Shimadzu, Tokyo, Japan). The wavelengths of fluorescence detection were 360 nm for excitation and 440 nm for emission. The mobile phase was methanol-water (45:55, vol/vol), and the flow rate was 1 mL/min.

### 4.6. Statistical Analysis

All data were analyzed according to a completely randomized experimental design, using the GLM procedure of SAS software (Version 9; SAS Institute, Inc., Cary, NC, USA). Duncan’s multiple range test was used for multiple comparisons when a significant difference was detected. Contrasts were performed to test the difference between the means of the moldy diet without and with *B.* *subtilis* ANSB060 (M0 *vs.* M0.5+M1.0+M2.0). Polynomial orthogonal contrasts were used to determine linear and quadratic responses to *B. subtilis* ANSB060 supplementation in the moldy diet. All statements of significance were based on the <0.05 level of probability.

## 5. Conclusions

In conclusion, our experiment demonstrates that the presence of aflatoxins in diets at a total level of 90.2 μg/kg (AFB_1_ = 70.7 μg/kg) could induce oxidative damage and excessive lipid peroxidation in the serum and liver, hepatocyte injury, liver tissue lesions, and serum biochemical changes in broilers. *B. subtilis* ANSB060 significantly decreased the amount of aflatoxins recovered from duodenum and suppressed the deleterious effects of aflatoxins on hepatic functions. The ameliorative effects were associated with the concentration of *B. subtilis* ANSB060 supplemented in the diet. Hence, *B. subtilis* ANSB060, as a feed additive for biodegradation of aflatoxins, may have promising potential in feed industrial applications.
